# Sports participation and sex outweigh the relative age effect in motor competence of school-aged children

**DOI:** 10.3389/fspor.2025.1608680

**Published:** 2025-09-19

**Authors:** Fábio Flôres, Denise Soares, Ana Filipa Silva, Nuno Casanova, Gabriela Almeida, José Marmeleira, Dimitar Shabanliyski

**Affiliations:** ^1^ Universidade de Évora, Escola de Ciências Sociais, Évora, Portugal; ^2^ Universidade de Évora, Centro de Investigação em Educação e Psicologia (CIEP), Évora, Portugal; ^3^ Universidade de Évora, Comprehensive Health Research Centre (CHRC), Évora, Portugal; ^4^ Liberal Arts Department, American University of the Middle East, Egaila, Kuwait; ^5^ Sport Physical Activity and Health Research & Innovation Center, Rio Maior, Portugal; ^6^ Insight: Piaget Research Center for Ecological Human Development, Instituto Piaget, Almada, Portugal; ^7^ Universidade de Évora, Escola de Saúde e Desenvolvimento Humano, Departamento de Desporto e Saúde, Évora, Portugal

**Keywords:** relative age effect, youth, performance, sports, education

## Abstract

**Background:**

The Relative Age Effect (RAE) refers to the advantage in physical and psychological development that children born earlier in the year often experience, which can influence their participation in sports.

**Aim:**

Analyze the influence of RAE on motor competence (MC) in school-aged children, focusing on differences across birth quartiles and types of sports participation.

**Methods:**

A cross-sectional study was conducted with 1,031 children aged 12.02 ± 2.95 years, stratified by birth quartile (Q1–Q4) and sports participation. MC was assessed using the Motor Competence Assessment (MCA) test battery. Statistical analyses included one-way ANOVA and a three-way MANCOVA.

**Results:**

Participants born in Q1 exhibited significantly higher MC scores compared to those born in Q3 and Q4, particularly in the Manipulative domain (*p* < 0.05, *η*^2^ = 0.01). Sports participation had the strongest effect (*p* < 0.01, *η*^2^ = 0.10), with participants in team sports demonstrating higher MC across all domains. Boys outperformed girls in Manipulative skills (*p* < 0.01, *η*^2^ = 0.12). Furthermore, the interaction between birth quartile, sports participation, and sex was also significant (*p* = 0.02, *η*^2^ = 0.01), indicating that the influence of RAE on MC depends on additional contextual factors.

**Conclusions:**

While RAE had a statistically significant but small effect on MC, sports participation, particularly team sports, and sex may play more dominant roles. These findings underscore the importance of promoting equitable access to organized physical activity while considering the nuanced and context-dependent nature of the RAE.

## Introduction

In the selection process for future talent, birthdates are a standard criterion for categorizing athletes, and in many cases, athletes are grouped into age groups or even two-year age groups. Although it aims to organize tournaments and training practices more effectively, this strategy overlooks the age and developmental differences inherent in youth (1, 2). In this perspective, the existing literature is well documented, indicating that, within a given age range, children and adolescents who are older and those who are younger can exhibit significant differences in numerous aspects, including physical and psychological maturation (2–5). As a result, the older ones tend to have more performance advantages than their younger counterparts, being more likely to be selected (6, 7), remaining overrepresented at the senior level (4). Therefore, literature has identified this issue as the relative age effect (RAE) phenomenon. Barnsley (8) proposed that when children are grouped solely by age, there can be a difference of up to a year in age among them (for example, children born in January will have an 11-month advantage over those born in December of the same year).

The RAE has been observed in numerous team and individual sports, regardless of the athletes' sex (4, 6, 9). Recent investigations indicate that an athlete's birth can influence their chances of achieving elite status within sports (10), particularly in youth sports, where physical and psychological maturity can differ significantly among children born months apart (1). In addition, the RAE was found in many sports, such as in Olympic athletes (11), alpine skiing (12), track and field (13), basketball, rugby, football, and volleyball (4, 6, 9, 14), and during fitness tests (15). In fact, this phenomenon has been observed in other disciplines beyond human kinetics, such as mathematics, algebra, measurement, geometry, statistics, and probability (16), as well as reading literacy (17). Recent results also suggest that this effect can be observed in elite Paralympic athletes (14).

Despite the growing evidence on the RAE across different performance contexts, fewer studies have addressed how this phenomenon influences broader developmental domains such as motor competence (MC), which is foundational for lifelong engagement in physical activity and sport. This is particularly important in a societal context increasingly affected by sedentary lifestyles and associated declines in youth physical fitness and quality of life. Notwithstanding, there are few investigations in the literature that examine the effects of RAE on MC in youth (18, 19). The literature has consistently demonstrated that MC is associated with higher levels of physical activity, and that children with higher levels also exhibit higher sports participation (20–23). Another interesting fact is that older children tend to present higher MC levels than their younger peers (24). Therefore, a better understanding of how RAE influences MC may help mitigate early dropout from physical activity and sport, thereby promoting healthier developmental trajectories. Nevertheless, a clear gap remains regarding how RAE interacts with different types of sports engagement (team vs. individual) and non-participation, which may differently affect opportunities for motor development and active lifestyles. While it remains relevant to explore how RAE influences MC, scientific evidence suggests that its impact may be better understood when analyzed in conjunction with sports participation and sex.

The purpose of the present investigation was to examine the effect of the RAE on MC scores in children and adolescents engaged in team and individual sports, as well as those not involved in regular sports participation. Specifically, it explores how variations in birth quartiles (Q) influence MC across these groups. Thus, this research aims to provide new insights into talent identification regarding children and adolescents participating in individual and team sports, emphasizing the importance of addressing RAE through age-appropriate interventions to ensure equitable physical and motor development opportunities. Furthermore, we would like to better understand whether the type of sports participation (team vs. individual) could influence the RAE. Our central hypothesis is that participants born earlier in the year will demonstrate superior MC levels compared to their younger peers within the same age cohort due to the cumulative advantages of being relatively older. Additionally, we anticipate differences in MC levels between children and adolescents who participate in sports vs. those who do not.

## Materials and methods

### Sample

This cross-sectional study included participants selected by convenience sampling in Portugal between 2023 (March and April) and 2024 (March and April). *a priori* sample size estimation was conducted using G*Power v3.1.9.7 (Kiel University, Germany) (25) based on a MANOVA: Global Effects model. The calculation considered 24 groups (4 birth quartiles × 3 types of sports participation × 2 sex), 4 dependent variables (MC components), a small effect size [*f*^2^(V) = 0.015], significance level *α* = 0.05, and *β* = 0.95, resulting in a sample of 936 participants.

Participants for this study were recruited from 10 urban public schools in the central region of Portugal. Schools were invited to participate through direct communication with their board directors, who facilitated access for students. Information regarding participants' sports participation, health status, and relevant background details has been collected in advance through discussions with their physical education teachers. Additional details were obtained from their legal guardians for students whose information was incomplete or unavailable. This recruitment process ensured a diverse representation of students across different age groups and levels of sports participation. Therefore, the inclusion criteria required participants to be children or adolescents aged 6–17 years with no history of injuries or illnesses that could affect MC. None of the participants had developmental difficulties or medical conditions that could negatively influence test performance.

A total of 1,031 children and adolescents (512 boys and 519 girls, aged 12.02 ± 2.95 years) were included in the investigation. Four hundred sixty-nine (45.5%; 230 boys and 239 girls) did not participate in sports on a regular basis. Participants involved in individual sports were 291 (28.2%; 117 boys and 174 girls), while those engaged in team sports were 271 (26.3%; 165 boys and 106 girls). Participants were also stratified into age groups and subdivided according to their birth quartiles (Q1: January–March, Q2: April–June, Q3: July–September, Q4: October–December) to examine the RAE (Table 1 for further details).

**Table 1:** Sample characteristics.

Quartiles	Variables	Boys			Girls		
		*n*	M	SD	*n*	M	SD
Q1	Age (years)	123	11.40	2.23	119	12.35	2.95
January to March	Weight (kg)		42.20	14.53		41.70	13.07
	Height (m)		1.49	0.15		1.49	0.14
	BMI (kg/m^2^)		18.68	3.66		18.48	3.46
Q2	Age (years)	150	11.74	2.91	150	12.54	3.02
April to June	Weight (kg)		41.78	15.87		44.61	12.71
	Height (m)		1.48	0.17		1.50	0.13
	BMI (kg/m^2^)		18.33	3.40		19.31	3.34
Q3	Age (years)	119	11.97	3.00	118	12.36	3.43
July to September	Weight (kg)		44.19	15.37		41.97	13.37
	Height (m)		1.51	0.17		1.48	0.15
	BMI (kg/m^2^)		18.65	3.26		18.71	3.46
Q4	Age (years)	120	11.63	2.96	132	12.19	2.86
October to December	Weight (kg)		44.80	16.75		42.40	14.65
	Height (m)		1.51	0.19		1.47	0.15
	BMI (kg/m^2^)		18.98	3.61		19.03	3.62

BMI, body mass index.

### Instruments and procedures

The assessments were conducted in a controlled setting during the afternoon (testing was always performed in a 40 × 20 m gymnasium at 20 degrees Celsius without any external interference). The data were collected between January 2023 and May 2024. The test set was administered in groups of five participants per test, with each group being supervised by a physical education-trained examiner (3 years of experience in collecting data). All data collection was also overseen by one of the authors of this study (PhD in motor behavior with 15 years of experience in data collecting).

Before collecting data, participants engaged in a standardized 10-min warm-up consisting of joint mobility exercises and light running exercises, following procedures described in the literature (26, 27). A verbal explanation and a proficient demonstration of all tests were always provided before participants were tested. Additionally, all participants underwent a trial test and were instructed to perform to the best of their ability. No feedback was provided regarding the test results or skill performance.

Prior to the assessment, oral consent was obtained from the participants, and written consent from their legal guardians. The University Ethics Committee approved the research (Protocol: P02-S09-27.04.22), which was conducted following the Declaration of Helsinki guidelines (28).

### Motor competence assessment—MCA

The MCA consists of six tests, grouped into three main components: Locomotor [Standing Long Jump (SLJ) and Shuttle Run (SHR) tests], Stability [Shifting Platforms (SP) and Jumping Sideways (JS) tests], and Manipulative [Ball Kicking Velocity (BKV) and Ball Throwing Velocity (BTV) tests]. MC is determined by summing the scores of these three components. The MCA was administered according to the protocol proposed in the literature (24, 29–31), which has demonstrated high reliability across all models, with coefficients ranging from 0.999 to 1.000 (31). All tests were quantitative and product-oriented, with no indication of a ceiling effect. Before the test administration, participants completed a familiarization trial to ensure proper understanding and execution (32).

### Standing long jump (SLJ)

Participants were instructed to jump with maximal effort, starting with both feet together. The distance was measured (in centimeters) as the distance from the starting point to the location of the heel of the foot closest to the starting point after the jump. Each participant had three jumps, with the best performance used for data analysis.

### Shuttle run (SHR)

All participants ran at maximal speed toward a line placed 10 meters apart, picked up a wooden block, ran back, and put it beyond the starting line. Subsequently, they were required to run back to retrieve the second wooden block and carry it across the finish line. Each participant completed two runs; the fastest time was used for data analysis.

### Jumping sideways (JS)

In the JS, participants should jump sideways with both feet together over a wooden beam (60 cm in length, 4 cm high, and 2 cm wide) as quickly as possible for 15 s. Each correct jump scored one point, and only the best result from the two attempts was considered.

### Shifting platforms (SP)

Children were instructed to move sideways for 20 s using two wooden platforms (25 cm × 25 cm × 2 cm). Each successful transfer from one platform to the other was scored with two points (one point for each step—passing the platform and moving the body to the platform). Only the best score from the two attempts was considered.

### Ball kicking velocity (BKV) and ball throwing velocity (BTV)

The BKV test required children to kick a soccer ball (circumference, 64.0 cm; mass, 360.0 g) against a wall with maximum effort. The BTV test required subjects to use an overarm action to throw a size tennis ball (diameter, 6.5 cm; mass, 57.0 g) against a wall with maximum effort. The speed of each attempt (BKV and BTV tests) was measured in meters per second using a radar gun (Pro II STALKER radar gun). The fastest speed from the three attempts was used for data analysis.

### MCA calculation

Standardized values (*Z*-scores) were calculated for each test, then *t*-scores were computed [*t* = 50 + (10 × *Z*)]. The three MC components (Locomotion, Stability, and Manipulation) were calculated by summing the *t*-scores of the respective tests within each category. Subtraction was performed in the Locomotion component due to the specific nature of the task, which required an inverse scoring approach (SHR measured in seconds). Finally, the overall MC scores were calculated as the average of the Locomotion, Stability, and Manipulation (29, 33).

### Data analysis

A combination of descriptive, inferential, and multivariate analyses was performed. Descriptive statistics were calculated to characterize the variables, including means and standard deviations. The Kolmogorov–Smirnov test was employed to assess data normality, and the Levene test confirmed the assumption of homogeneity of variances (*p* > 0.05) (34). To explore differences in MC and its components across birth quartiles, sex, and sports participation, inferential analyses were conducted using one-way ANOVA. *post-hoc* Tukey's tests were performed for pairwise comparisons where significant group differences were identified. A three-way MANCOVA was conducted to examine the effects of birth quartile, sports participation, and sex on Locomotor, Stability, Manipulative, and MC. Birth quartile (Q1–Q4), sports participation (non-sports participation, individual and team sports), and sex (boys, girls) were included as fixed factors. The full factorial model tested the main effects, two-way interactions (birth quartile × sports participation, birth quartile × sex, sports Participation × sex), and three-way interactions (birth quartile × sports participation × sex). *post-hoc* Tukey's tests were applied where significant differences were found. Partial eta-squared (*η*^2^) values were reported to assess effect sizes. The Statistical Package for the Social Sciences (SPSS, IBM Corporation), version 29.0, was used, adopting an alpha significance level of 5%.

## Results

The descriptive statistics for MC divided by birth quartiles are shown in Table 2. Data is presented controlling for the MC by sex and sports participation. Descriptive statistics revealed that participants in team sports had the highest mean scores in all MC components, followed by those in individual sports. In contrast, non-sport participants consistently showed the lowest scores.

**Table 2:** Sample characteristics regarding percentiles of MC and its components.

Quartiles	Groups	MC variables	Boys			Girls		
			*n*	M	SD	*n*	M	SD
Q1	Non-sport participation	Locomotor	64	49.72	5.54	49	45.68	5.99
		Stability		48.55	6.76		45.28	6.77
		Manipulative		51.40	7.79		42.47	4.23
		MC		49.93	5.26		45.44	5.14
	Individual sports	Locomotor	19	50.15	5.25	49	50.42	8.63
		Stability		48.36	6.92		49.40	9.38
		Manipulative		52.96	7.31		46.91	7.35
		MC		50.12	5.91		50.49	7.42
	Team sports	Locomotor	40	50.18	8.82	21	50.98	4.95
		Stability		52.12	6.94		59.72	11.77
		Manipulative		55.81	8.73		52.97	6.19
		MC		52.42	9.49		53.59	5.94
Q2	Non-sport participation	Locomotor	75	47.80	8.77	80	46.42	7.69
		Stability		46.92	8.20		46.90	7.95
		Manipulative		50.08	7.08		43.09	5.46
		MC		47.73	8.34		45.75	7.42
	Individual sports	Locomotor	27	54.00	11.38	37	49.21	7.72
		Stability		51.09	10.54		49.18	8.27
		Manipulative		53.59	9.14		45.78	8.42
		MC		53.53	11.18		48.63	7.76
	Team sports	Locomotor	48	51.71	8.84	33	50.25	6.24
		Stability		53.96	8.69		56.02	9.98
		Manipulative		56.18	10.08		52.85	6.41
		MC		53.09	9.04		52.13	6.67
Q3	Non-sport participation	Locomotor	50	50.24	9.59	57	47.24	6.17
		Stability		47.30	9.64		47.69	6.50
		Manipulative		50.78	8.40		43.86	4.55
		MC		49.43	9.44		46.63	5.64
	Individual sports	Locomotor	32	49.94	13.70	45	45.29	8.42
		Stability		49.29	9.65		45.84	8.87
		Manipulative		50.75	9.67		42.26	7.83
		MC		50.56	10.29		44.59	8.48
	Team sports	Locomotor	37	48.44	8.66	16	51.29	4.76
		Stability		49.91	8.84		59.28	10.81
		Manipulative		52.36	7.64		52.08	3.95
		MC		49.27	8.73		53.56	4.93
Q4	Non-sport participation	Locomotor	41	45.92	6.98	53	45.42	7.26
		Stability		45.66	8.35		45.47	7.06
		Manipulative		47.44	6.13		43.36	4.56
		MC		44.86	5.78		44.75	6.72
	Individual sports	Locomotor	39	57.22	10.80	43	47.35	8.71
		Stability		53.93	10.65		45.44	8.88
		Manipulative		54.82	12.12		41.63	6.35
		MC		55.25	10.19		45.79	8.33
	Team sports	Locomotor	40	48.95	11.18	36	51.34	7.61
		Stability		48.60	8.96		55.91	12.30
		Manipulative		54.53	8.31		52.33	9.27
		MC		48.77	8.92		52.77	8.10

MC, motor competence.

The results are reported in *t*-scores, a standardized metric with a mean of 50 and a standard deviation of 10.

**Figure 1:**
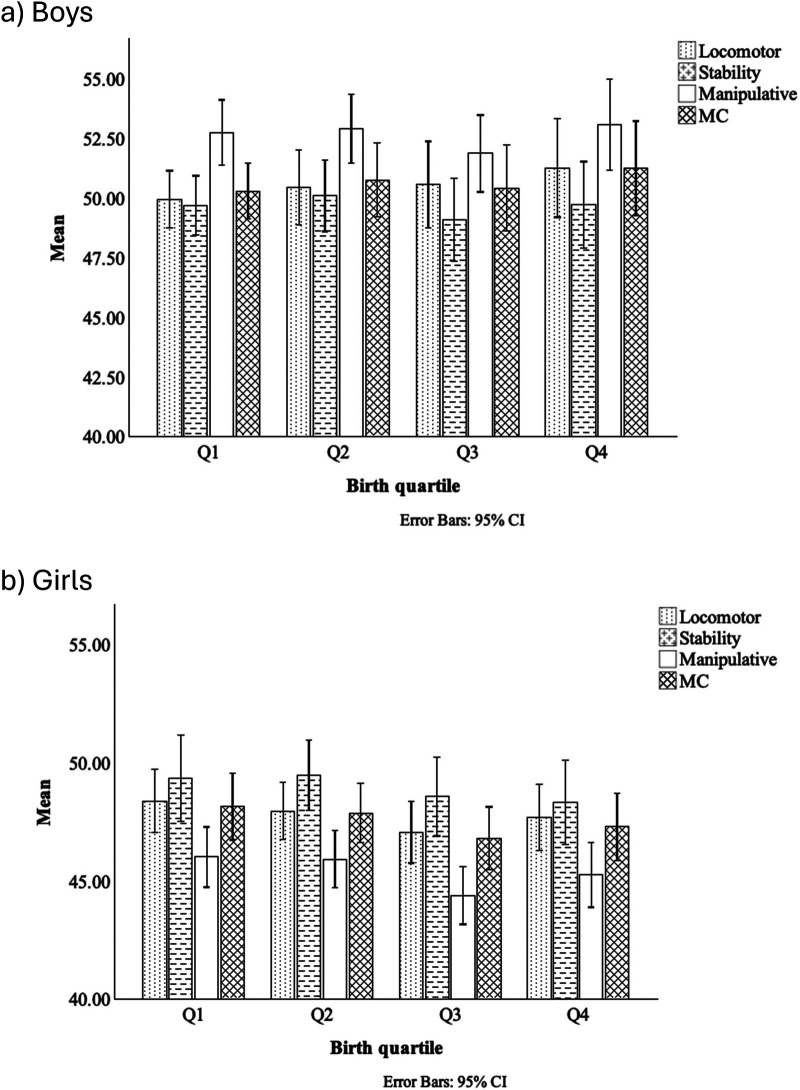
Mean and standard deviation for MC across birth quartiles, according to sex: **(a)** boys; **(b)** girls. Q1: January to March; Q2: April to June; Q3: July to September; Q4: October to December. *Q1 > Q3, *p* < 0.05.

General one-way ANOVA results indicate statistically significant differences between birth quartiles only in the Manipulative and MC domains. In the Manipulative category, a significant difference is observed between Q1 and Q3 (*p* = 0.02; *η*^2^ = 0.01). In MC, significant differences are found between Q1 and Q3 (*p* = 0.03; *η*^2^ = 0.01) and between Q1 and Q4 (*p* = 0.04; *η*^2^ = 0.01). Controlling for data by sex (Figure 1), no statistically significant differences were found in boys' Locomotor (*p* = 0.87; *η*^2^ = 0.00), Stability (*p* = 0.69; *η*^2^ = 0.00), Manipulative (*p* = 0.42; *η*^2^ = 0.01), and MC (*p* = 0.64; *η*^2^ = 0.00) (Figure 1a). The same patterns were also found in girls for the Locomotor (*p* = 0.48; *η*^2^ = 0.01), Stability (*p* = 0.66; *η*^2^ = 0.00), and Manipulative (*p* = 0.23; *η*^2^ = 0.01). However, in MC, there is a significant difference between Q1 and Q3 (*p* = 0.03; *η*^2^ = 0.01) (Figure 1b), indicating a small but meaningful effect of birth quartile on MC in this category.

**Figure 2:**
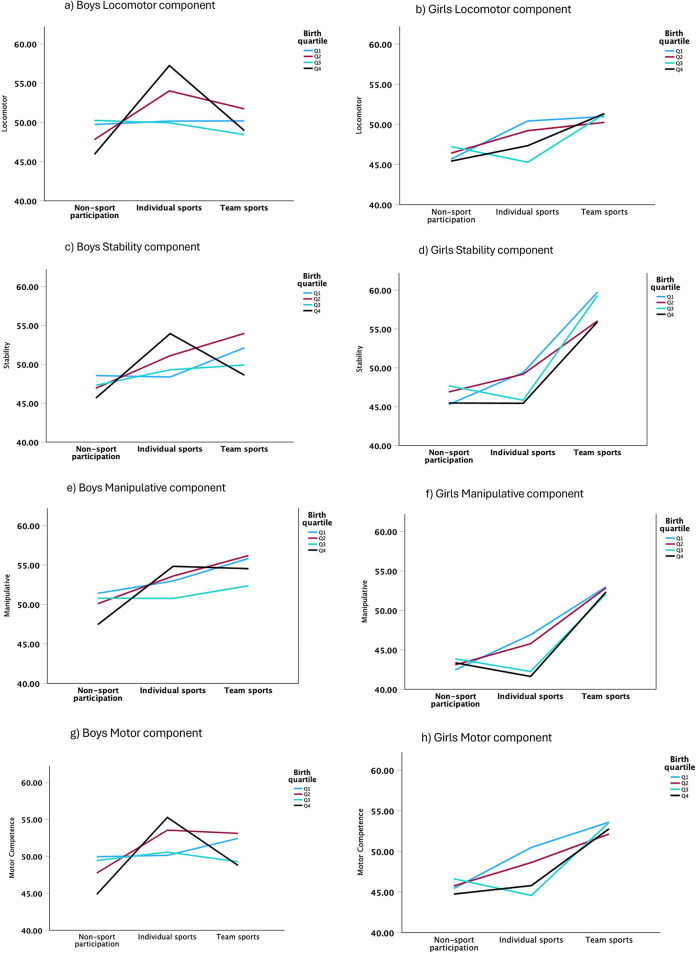
MC components scores (Locomotor, Stability, Manipulative, and Motor) according to sex (**a**, **c**, **e**, **g** for boys; **b**, **d**, **f**, **h** for girls) sports participation (non-sport, individual, or team sports), and birth quartiles (Q1: January to March; Q2: April to June; Q3: July to September; Q4: October to December).

The MANCOVA revealed significant main effects of birth quartile, sports participation, and sex on MC (Wilks' Lambda < 0.05, *p* < 0.01), indicating that these factors influence MC levels. Sports participation had the strongest multivariate effect (Wilks' Lambda = 0.81, *F* = 27.67, *p* < 0.01, *η*^2^ = 0.10), with team sports participants consistently scoring higher in all MC components than individual and non-participants in sports. Sex also had a significant effect (Wilks' Lambda = 0.79, *F* = 67.15, *p* < 0.01, *η*^2^ = 0.21), with boys demonstrating higher scores overall, particularly in Manipulative skills (*F* = 137.90, *p* < 0.01, *η*^2^ = 0.12). However, the sex effect was non-significant for Stability (*p* = 0.14). Birth quartile had a multivariate significant impact (Wilks' Lambda = 0.95, *F* = 4.11, *p* < 0.01, *η*^2^ = 0.02); however, univariate analyses revealed that its impact was small and inconsistent across MC components. A significant effect was found for the Manipulative component (*F* = 2.99, *p* = 0.03, *η*^2^ = 0.01) but not for the Locomotor (*p* = 0.53) or the Stability (*p* = 0.20). The interaction between birth quartile and Sports Participation was significant for the Locomotor component (*F* = 3.43, *p* = 0.00, *η*^2^ = .02) and MC (*F* = 2.56, *p* = 0.02, *η*^2^ = 0.02), suggesting that the effects of birth quartile on MC are influenced by sports participation (Figure 2).

A significant finding was the three-way interaction among birth quartile, sports participation, and Sex (Wilks' Lambda = 0.96, *F* = 1.79, *p* = 0.02, *η*^2^ = 0.01). The interaction effects were significant in the Locomotor component (*F* = 2.97, *p* = 0.01, *η*^2^ = 0.02), Stability (*F* = 3.08, *p* = 0.01, *η*^2^ = 0.02), and Manipulative (*F* = 2.46, *p* = 0.02, *η*^2^ = 0.01), indicating that the relationship between birth quartile and sports participation varies according to sex. While team sports participation benefits both boys and girls, boys tend to score higher in all birth quartiles, particularly in the Manipulative component. In contrast, girls' scores vary more depending on the type of sport. These findings highlight that sports participation plays a significant role in MC development, with team sports showing the most significant positive impact in our sample. While RAE alone has limited influence, its effects become more apparent when considering sports participation and sex differences.

## Discussion

The present study aimed to analyze the impact of the RAE on MC in school-aged children and adolescents, with a specific focus on how variations in birth quartiles and sport type influence their MC. While RAE showed a statistically significant influence on MC, its effect was limited in magnitude. Results showed that participants born in the first half of the year (Q1 and Q2) had significantly higher MC scores than those born between July–September (Q3) and October–December (Q4), although the effect was not large (*η*^2^ = 0.01), which is in agreement with existing literature (1, 6, 35). For example, in a systematic review, Cobley et al. (1) identified a consistent prevalence of RAEs, albeit with small effect sizes, across 253 independent samples from 14 sports and 16 countries. Morganti et al. (4) found that early-born players (January to March) were significantly overrepresented in youth national teams, particularly in the UEFA U17 European Championship, where teams with stronger RAEs tend to achieve better results. Findings also showed that at the senior level, RAEs persist but weaken, with a relative increase in later-born players (BQ4), suggesting a reversal effect where initially disadvantaged players develop resilience and long-term potential. The author discussed that their results highlight the inefficiency of early selection systems that prioritize short-term success over long-term talent development, calling for systemic reforms in player identification and development strategies. Nevertheless, our results also showed that sports participation, especially in team sports, and sex differences played a much stronger role in MC outcomes.

The most pronounced differences in our general sample were in the Manipulative component, where participants born in Q1 scored significantly higher than those born in Q3. This is consistent with previous research suggesting that Manipulative skills, such as ball control and object manipulation, may be more influenced by relative age advantages due to increased exposure and practice opportunities (24). However, the effect of birth quartile was not significant for the Locomotor or Stability components, suggesting that other factors, such as overall physical activity and other extracurricular activities, may play a greater role in these components.

Another finding is the observation that, overall, MC was significantly different across birth quartiles for girls. At the same time, none of the individual components (i.e., Locomotor, Stability, Manipulative) showed significant differences. One plausible explanation is that although each motor domain shows only a marginal or non-significant effect in isolation, the cumulative small effects across all three domains may have contributed to a significant difference in the overall MC score. This combination effect is particularly relevant when variability is low within each component but consistent across domains, which appears to be the case in this subgroup. In addition, the finding that manipulative skills were significantly higher in Q1 compared to Q3 (36), but not Q4, may be attributed to the variability in sports participation. It is possible that children born in Q4 benefited from compensatory mechanisms, such as increased participation in structured physical activities or higher motivation due to awareness of early disadvantage (37, 38). These mechanisms have been proposed in the literature as potential buffers against RAE in later-born children, referred to as the “underdog hypothesis”. Towlson et al. (10) suggested that children born earlier in the year tend to show more advanced physical and psychological development, which can enhance their sports performance and motor skills. The findings concerning RAE influence in the Stability and Manipulative components are more pronounced than in the Locomotor component, corroborating previous studies that emphasize the sensitivity of specific motor skills to maturity differences (24, 29). The RAE hypothesis also suggests that children and adolescents born earlier in the selection period (e.g., school year) tend to have an advantage in MC because they are older and more physically developed than their younger peers (39). This advantage can also lead to higher participation rates in sports and better MC scores.

Regarding sports participation, our results demonstrated that participants engaged in team sports consistently outperformed both individual sports participants and non-sport participants across all MC components. The MANCOVA results indicated that sports participation had the strongest multivariate effect on MC, indicating the considerable role of organized sport in MC development. These findings reinforce the importance of sports environments in enhancing motor behavior and align with previous investigations emphasizing the benefits of team sports participation (33, 40).

The differences observed between team sports and individual sports suggest that social and environmental factors also play a role in MC development. Team sports participants are involved in more dynamic interactions, requiring athletes to adapt quickly to unpredictable situations, which may contribute to enhanced motor adaptability and coordination. Additionally, the frequent need for teamwork, passing, and defensive positioning in team sports fosters a broader range of movement patterns and cognitive decision-making skills compared to individual sports (41). In contrast, individual sports tend to emphasize self-paced, repetitive movements, which, while beneficial for specific skill development, may not provide the same level of variability and adaptability. This could explain why team sports participants demonstrated higher MC scores across all measured components, as their training environments likely encourage a more holistic development of MC. Despite this, factors other than the type of sport should be analyzed mainly in younger age categories, such as the under-13 category, where relatively older participants show better performance due to early exposure to formal competition (42).

Additionally, previous research has demonstrated that RAE also affects fundamental movement skills in non-sporting contexts (19, 43, 44). For example, children born in the first quarter of the year exhibit higher proficiency in skills like catching and throwing compared to those born later (43) and perform better in MC assessments, including manual dexterity and balance, compared to their peers born in the second semester (45). Children with low MC are less likely to participate in physical activities and sports, further delaying their motor development (44, 46). This creates a cycle where low MC leads to less participation, which prevents improvement in motor skills, supporting Stodden et al. (47) assumptions. Also, children's perception of their MC can influence their willingness to participate in physical activities (48). Over time, children who accurately perceive their motor abilities are more likely to engage in physical activities, which can improve their MC.

Sex differences were also evident, with boys exhibiting significantly higher MC scores overall, particularly in the Manipulative component (*F* = 137.90, *p* < 0.01, *η*^2^ = 0.12), confirming previous findings (24). While sex differences were non-significant for Stability, boys generally performed better in Manipulative and Locomotor tasks. This finding is consistent with prior research indicating that boys tend to develop greater upper-body strength and coordination, which contributes to their superior performance in object control tasks (2, 29, 49). A notable finding was the significant interaction effect between birth quartile, sports participation, and sex (Wilks' Lambda = 0.96, *F* = 1.79, *p* = 0.02, *η*^2^ = 0.01), suggesting that the relationship between RAE and MC varies according to both sport type and sex. While boys consistently demonstrated higher MC scores across all quartiles, girls' performance was more variable, particularly in team sports. This finding highlights the intricate relationship between relative age, sport type, and sex in MC development, underscoring the need for further investigation.

It is essential to consider how the team sport environment may interact with mechanisms that attenuate or amplify the RAE. Team sports often involve frequent exposure to competitive situations, peer comparison, and conditions that may intensify the advantages of relatively older participants (50). However, these same conditions may also foster adaptive mechanisms in relatively younger players. For instance, later-born individuals may develop superior self-regulation, tactical awareness, and psychological resilience, enabling them to remain competitive in team settings where they face maturational disadvantages (37). Our data supports this possibility, as children in team sports, regardless of birth quartile, showed higher MC scores overall, suggesting that the structured and dynamic nature of team sports may create an environment that not only expands RAE-related disparities but also offers pathways for their mitigation through skill-based compensation, role flexibility, and social reinforcement (51). These mechanisms warrant further investigation but provide a meaningful explanation for why team sport participation emerged as the strongest predictor of MC in our study.

Previous investigations corroborate these findings, where both boys and girls born in the first semester of the year tend to have higher MC scores compared to those born in the second semester (45, 52). Age was also identified as a significant covariate for boys but not girls. Studies have shown that older children generally perform better in various motor tasks than their younger peers in the same age group (49, 53). In the case of boys, this can be explained by the motor skills that tend to improve with age due to increased physical and cognitive development (2, 49), becoming a factor that can influence the RAE in older ages. Our results indicate that younger participants (e.g., elementary school students) show significant RAE, but the effect may diminish with age (54). The literature also indicates that older players (Q1 and Q2), born earlier in the year, are overrepresented in sports, suggesting that they are more likely to be selected and participate at higher levels (55). For example, in under-18 girls' volleyball, a higher percentage of medalist athletes were born at the beginning of the year, indicating an advantage for those with earlier birthdates (56).

Contrary to some of the studies mentioned earlier, our results indicate that the RAE alone has a limited impact on MC when additional factors, such as sports participation and sex differences, are not considered. While participants born earlier in the year exhibited slightly higher MC scores, the magnitude of these differences was small, reinforcing that participation in organized physical activities also plays a decisive role in MC development, more so than RAE alone.

Despite our important results, some limitations need to be highlighted. First, this study employs a cross-sectional design, which does not allow us to establish causality between RAE and MC. Although the statistical analysis controls for factors such as age and sex, it does not account for potential confounding variables like socioeconomic status, access to sports facilities, other extracurricular activities, or regional differences. Additionally, it focuses on participants within continental Portugal, which may limit the applicability of the findings to different cultural or geographical contexts. Future research should consider longitudinal designs, diverse populations, age ranges, and mixed-method approaches to address these limitations comprehensively​. Additionally, investigations analyzing RAE during individual and team sports, as well as those examining cross-cultural differences, may help to better understand this phenomenon.

## Conclusion

The present investigation highlights the influence of RAE, sports participation, and sex in MC among school-aged children and adolescents. While children born earlier in the year tend to demonstrate higher MC scores, the effect is relatively small and primarily observed in the Manipulative component. Sports participation, particularly in team sports, has a much more pronounced influence on MC development, emphasizing the importance of providing opportunities for all participants to engage in structured physical activities. Schools and sports programs should personalize teaching approaches to account for RAE. Monitoring MC, adjusting assessment criteria, and implementing fair selection processes can help balance advantages among children of different birth quartiles. Ensuring equitable opportunities prevents early dropouts and promotes long-term engagement and motivation in physical activity. Therefore, our results strongly suggest that by adapting practices, educators, coaches, and policymakers can help create environments where children and adolescents succeed, regardless of their birth quartile.

## Data Availability

The raw data supporting the conclusions of this article will be made available by the authors, without undue reservation.
